# Myeloid hypoxia-inducible factor HIF1A provides cardio-protection during ischemia and reperfusion *via* induction of netrin-1

**DOI:** 10.3389/fcvm.2022.970415

**Published:** 2022-09-28

**Authors:** Ka Lin Heck-Swain, Jiwen Li, Wei Ruan, Xiaoyi Yuan, Yanyu Wang, Michael Koeppen, Holger K. Eltzschig

**Affiliations:** ^1^Department of Anesthesiology and Intensive Care Medicine, Tübingen University Hospital, Eberhard Karls University of Tübingen, Tübingen, Germany; ^2^Department of Anesthesiology, McGovern Medical School, The University of Texas Health Science Center at Houston, Houston, TX, United States; ^3^Department of Cardiac Surgery, Sir Run Run Shaw Hospital, School of Medicine, Zhejiang University, Hangzhou, China; ^4^Department of Anesthesiology, Second Xiangya Hospital, Central South University, Changsha, China

**Keywords:** hypoxia, myocardial infarction, polymorphonuclear neutrophil (PMN), reperfusion, heart, hypoxia inducible factor (HIF)

## Abstract

The transcription factor hypoxia-inducible factor HIF1A induces cardioprotection from ischemia and reperfusion injury. Here, we investigate tissue-specific pathways that are critical for HIF1A-elicited tissue protection. Initial studies showed that mice with induced global Hif1a deletion (*Hif1a^loxP/loxP^* UbiquitinCre+) have exaggerated myocardial injury during *in situ* ischemia and reperfusion. Surprisingly, this phenotype was mirrored only in mice with myeloid-specific *Hif1a* deletion *(Hif1a*^loxP/loxP^** LysM Cre+). In contrast, mice with myocardial specific (*Hif1a^loxP/loxP^* Myosin Cre+), or vascular *Hif1a* deletion (*Hif1a^loxP/loxP^* VEcadherin Cre+) experienced similar levels of injury as controls. Subsequent studies using adoptive transfer of *Hif1a-*deficient polymorphonuclear neutrophils (PMNs) prior to myocardial injury demonstrated increased reperfusion injury. On the contrary, the adoptive transfer of PMNs treated *ex vivo* with the hypoxia inducible factor (HIF) stabilizer dimethyloxalylglycine (DMOG) was associated with attenuated myocardial injury. Furthermore, DMOG-mediated cardioprotection was abolished in *Hif1a^loxP/loxP^* LysM Cre+ mice, but not in *Hif2a^loxP/loxP^* LysM Cre+ mice. Finally, studies of PMN-dependent HIF1A target genes implicated the neuronal guidance molecule netrin-1 in mediating the cardioprotective effects of myeloid HIF1A. Taken together, the present studies identified a functional role for myeloid-expressed HIF1A in providing cardioprotection during ischemia and reperfusion injury, which is mediated, at least in part, by the induction of the netrin-1 neuronal guidance molecule in neutrophils.

## Introduction

Myocardial infarction remains one of the leading causes of death worldwide. Most commonly, it is an aftereffect of a coronary artery occlusion leading to myocardial ischemia (1). As a consequence of ischemia, myocardial tissue develops profound hypoxia (2), leading to tissue necrosis if blood flow is not restored in time. During myocardial ischemia, tissue hypoxia becomes a strong transcriptional stimulus that activates a coordinated transcriptional program that promotes increased resistance to ischemia and reperfusion injury (3, 4). A central role in the coordination of this adaptive response is mediated by the stabilization of the hypoxia-inducible transcription factor HIF1A (5).

Under normoxic conditions, HIF1A is constantly degraded through the proteasomal pathway due to hydroxylation by prolylhydroxylases (PHDs), causing subsequent binding of the von Hippel Lindau gene product, which targets HIF1A for degradation (6, 7). However, when oxygen levels drop, PHDs become inactivated and HIF1A is rapidly stabilized, translocates to the nucleus, and binds to the promoter region of hypoxia inducible factor (HIF) target genes to induce their transcription (2, 3). HIF1A is known to regulate a wide array of genes. For example, gene expression profiles in endothelial cells exposed to normoxia or hypoxia indicate that more than 600 target genes are regulated by HIF1A, including gene repression or induction (8). In cardiomyocytes, HIF1A increases the expression of glycolytic pathway enzymes, thereby enhancing their capacity to generate ATP under anaerobic conditions and promoting their ability to cope with ischemic tissue injury (9). In myeloid cells, HIF1A transcriptional activity can regulate cell functions or drive the release of cardioprotective HIF1A target genes (10–12), which can significantly influence inflammatory processes (13). For example, stabilization of HIF1A in hypoxia increases the life span of usually short-lived polymorphonuclear neutrophils (PMN) (14). Furthermore, stabilization of HIF1A increases the motility of myeloid cells toward a chemotactic gradient, bringing them to the site of injury (15, 16).

Based on studies implicating HIF1A stabilization in tissue adaptation and protection during hypoxia or inflammation (3, 17, 18), research efforts were initiated to use this pathway as a therapeutic target. Several pharmaceutical companies have been successful in finding small molecule inhibitors for PHDs that can be used as HIF activators. In fact, several recent phase-3 clinical trials successfully tested the PHD inhibitors roxadustat or vadadustat for the treatment of renal anemia (19–22). Furthermore, experimental studies provide evidence that PHD inhibitors induce cardioprotection from ischemia and reperfusion injury (5, 23). Together, these studies highlight that the HIF1A pathway could be utilized as a therapeutic target for myocardial ischemia and reperfusion injury.

Cardioprotection by HIF1A is supported by several previous studies (24–26). However, the cellular source of HIF-mediated cardioprotection remains unknown. HIF1A is ubiquitously expressed, including cells involved in myocardial ischemia and reperfusion injury, such as cardiomyocytes, vascular endothelial cells, and myeloid cells (23, 27). To uncover the individual contributions of different cellular compartments during HIF1A-dependent cardioprotection, we used transgenic mice with a *Hif1a* gene flanked by a LoxP site. When breeding these mice with tissue-specific expression of Cre recombinase, we stepwise deleted *Hif1a* from different cellular compartments. Surprisingly, we found that the predominant source of HIF1A-mediated cardioprotection involves myeloid cells and uncovered a novel contribution of PMN.

## Materials and methods

### Mice

All animal procedures were performed in an accredited facility of the American Association for Laboratory Animal Care and approved by the University of Colorado Denver and the Institutional Animal Care and Use Committee of the University of Texas Health Science Center at Houston (UTHealth). For all studies, we used mice with an age of 8–16 weeks. C57BL/6J mice were purchased from The Jackson Laboratory (Bar Harbor, ME, United States). *Hif1a^loxP/loxP^* (B6.129-*Hif1a*^*TM*3*Rsjo*^/J) (28), *Hif2a^loxP/loxP^* (Epas1tm1Mcs/J) (23), *Ntn1^loxP/loxP^* (B6.129(SJL)-Ntn1tm1.1Tek/J) (12). These animals were crossed with the following mouse lines, that express Cre-recombinase under the control of a tissue-specific promoter: tamoxifen-inducible Ubiquitin Cre+ (B6.Cg-Ndor1Tg (UBC-cre/ERT2) 1Ejb/J) (29), LysM Cre+ (B6.129P2-Lyz2TM 1 (cre)Ifo/J) (30), VE-cadherin-Cre+ (B6.Cg-Tg(Cdh5-cre)7Mlia/J) (31, 32), and tamoxifen inducible Myosin-Cre+ (B6.FVB(129)-A1cfTg). For studies using Myosin-Cre+ or Ubiquitin Cre+ mice, mice received tamoxifen dissolved in peanut oil for 5 days (1 mg/day i.p.), followed by a resting period of 7 days prior to experimentation. Mice were genotyped by GeneTyper (New York, NY, United States) or in house according to the recommended protocol.

### Human neutrophil isolation and cell culture

From healthy subjects, human blood was collected in tubes containing anticoagulant citrate (S-monovette #04.1902, Sarstedt, Nümbrecht, Germany). Neutrophils were isolated using a Percoll-based protocol. Briefly, whole blood was applied to the discontinuous Percoll gradient containing equal vols of 63 and 72% Percoll solution and centrifuged at 400 (× *g*) for 30 min without brake. A mixture of neutrophils and red blood cells was collected at the Percoll gradient interface, in which the latter were subsequently removed by isotonic lysis (NH_4_Cl solution). Neutrophils were then pelleted and washed with HBSS(-). Neutrophil purity/viability was assessed by loading a small aliquot into a particle count and size analyzer (Coulter, Backman Coulter) following the manufacturer’s instructions. In total, 1–2 × 10^6^ PMN per ml of blood were typically isolated with >95% purity and viability. After isolation, neutrophils were rested 30 min before experiments. Cells were cultured 2.5 × 10^6^ cells/ml in RPMI 1640 with 10% FCS at 37°C in 21% O_2_ (0 h timepoint) or 2% O_2_ (hypoxia timepoints).

### Murine model for myocardial ischemia

A detailed procedure of *in situ* myocardial ischemia and reperfusion injury has previously been described (23, 33–35). In summary, we exposed mice to 60 min of myocardial ischemia followed by 2 h of reperfusion, followed by an analysis of the size of the infarct and a measurement of cardiac troponin I (cTnI) in serum. Dimethyloxalylglycine (DMOG) was administered to mice 4 h before ischemia started (1 mg/mouse, dissolved in normal saline, intraperitoneal). Successful occlusion of LCA occlusion was confirmed by a color change in LCA-supplied cardiac tissue. After 60 min of ischemia, the weights were removed to allow reperfusion of the tissue. During ischemia, we administered normal saline at a constant rate of 0.1 ml/h. At the beginning of reperfusion, we increased the infusion rate to 0.6 mL/h.

### Infarct staining

The detailed procedure to measure the percentage of infarcted area relative to the area undergoing ischemia (area at risk) using a counterstain technique was previously described (23, 33). At the end of the experiment, the circulation was flushed with 5 ml of normal saline and LCA was permanently occluded. After injection of Evan’s blue dye (600 μl, 1%), the heart was excised by the heart basis and kept at −20°C for 15 min. The hearts were then cut and incubated with 1% triphenyltetrazolium chloride (TTC) for 10 min at 37°C before fixation in 4% neutral buffer formalin. To prevent bleaching of the dye, photographs of the heart slices were taken the day after the experiments. For this, tissue slices were placed between glass slides and pictures were taken using a Nikon D5300 camera at 32× magnification. The AAR and infarct size were measured by ImageJ software (National Institutes of Health, United States; Version 1.51k). If an air bubble was accidentally injected into the cardiac circulation during Evan’s blue injection, resulting in incomplete delimitation of AAR, the sample was excluded.

### Cardiac troponin I enzyme-linked immunosorbent assay

As an additional line of evidence for myocardial injury, cTnI was measured by enzyme-linked immunosorbent assay (ELISA), as described (36) using the cTnI ELISA Kit (Life Diagnostic, #CTNI-1-HS).

### Neutrophil depletion and adoptive transfer

Untouched PMNs were isolated from bone marrow in donor mice of the indicated genotype (aged 6–8 weeks) using the EasySep mouse neutrophil enrichment kit (Stemcell Technologies Inc.). In a subset of the experiment PMN from C57/J animals, 1 mM DMOG (Sigma-Aldrich, #D3695) or vehicle solution was incubated for 60 min. Then 1 × 10^6^ cells were injected into neutrophil-depleted mice through an arterial catheter over a 10-min period 60 min prior to myocardial ischemia. The detailed procedure for neutrophil depletion *in vivo* was previously described (37) using Ly6G-specific mAb 1A8 antibody (BioXCell).

### Immunoblotting experiments

To study the expression of Netrin-1, immunoblotting methods were conducted. At the end of each timepoint, neutrophils in the medium were collected and centrifuged at 500 (× *g*) for 5 min to collect cells. Cells were immediately lysed in IP lysis buffer (Pierce #87788, ThermoFisher) on ice for 30 min and then centrifuged at 12,000 (× g) for 10 min. The protein concentration of the lysates was measured by the BCA Assay (#23225, ThermoFisher) then incubated in Laemmli sample buffer containing 2-mercaptoethanol at 95°C for 5 min. Lysate proteins (40 μg) were separated on 7.5% SDS polyacrylamide gels by SDS-PAGE and then transferred to PVDF membranes using a Bio-Rad Mini Trans-Blot cell. Netrin-1 (LSBio, LS-C743016) and β-actin (Santa Cruz, sc-130656), used as loading control, were detected using specific primary antibodies. The membranes were subsequently incubated with a secondary antibody coupled to HRP conjugate (Invitrogen #65-6120). The membranes were incubated with ECL reagents (Clarity Max, Bio-Rad, and Immunocruz, Santa Cruz, respectively) and the chemiluminescent signal detected by the Fusion Imaging System (Vilber Lourmat). Protein expression was quantified by densitometry using ImageJ (National Institutes of Health, United States; Version 1.51k) and normalized to the expression of β-actin.

### Statistical analysis

The N numbers were predetermined by power analysis to detect a mean difference of 20% with 8% standard deviation (SD). Outliers were detected by the Grubb test and removed from the data. All data approximately followed the normal distribution and were plotted as mean ± SD. The experimental data were analyzed by the unpaired two-sided student *t*-test or ANOVA with Tukey test for multiple comparison. A *p*-value less than 0.05 was considered statistically significant. Statistical analyzes were performed using Prism (version 9.1.2, GraphPad Software Inc.).

## Results

### Global-induced deletion of Hif1A is associated with increased myocardial ischemia and reperfusion injury

**FIGURE 1:**
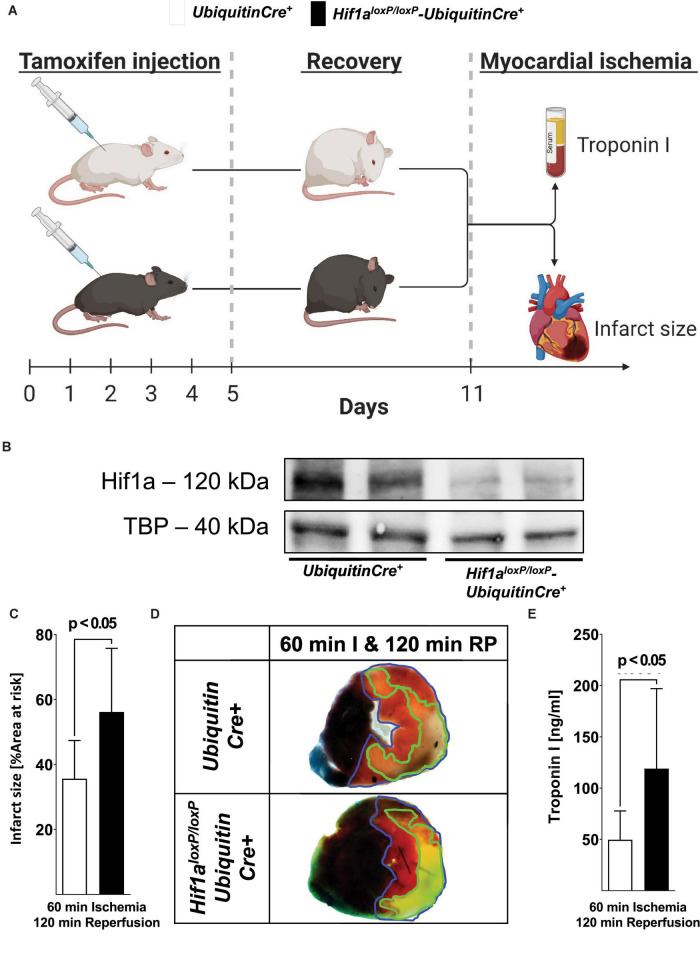
Functional consequences of the induced global deletion of *Hif1a* during myocardial ischemia and reperfusion injury. **(A)** Experimental approach to study global induced Hif1a-deficiency. *Hif1a^loxP/loxP^* mice were crossed with Cre-recombinase expressing mice under the control of a ubiquitin promoter (UbiquitinCre+); these mice express cre-recombinase under the control of a tamoxifen inducer. Control animals with sex and weight matched (UbiquitinCre+) or *Hif1a^loxP/loxP^* UbiquitinCre+ mice received a daily dose of 1 mg i.p. tamoxifen 5 consecutive days to induce Cre recombinase activity. After 7 days, the animals underwent experimental protocols (60 min of *in situ* myocardial ischemia followed by 120 min of reperfusion), followed by serum collection to determine the concentrations of troponin I and TCC staining for infarct sizes. **(B)** Hif1A immunoblot performed on protein isolated from myocardial tissue after treatment with the pharmacological HIF activator dimethyloxalylglycine (1 mg DMOG i.p.) 4 h before tissue collection from UbiquitinCre+ or *Hif1a^loxP/loxP^* UbiquitinCre+. A representative image of three individual experiments is presented. **(C)** The infarct sizes of UbiquitinCre+ or *Hif1a^loxP/loxP^* UbiquitinCre+ mice are displayed as a percentage of the area at risk after 60 min of ischemia, followed by 120 min of reperfusion (UbiquitinCre+ *n* = 7; *Hif1a^loxP/loxP^* UbiquitinCre+ *n* = 5, per group mean ± SD; statistical analysis by two-sided, unpaired Student’s *t*-test; mice were matched by age, sex, and weight). **(D)** Representative infarct staining of UbiquitinCre+ or *Hif1a^loxP/loxP^* UbiquitinCre+ mice (60 min of ischemia and 120 min of reperfusion). **(E)** Troponin serum levels after 60 min of ischemia, followed by 120 min of reperfusion in UbiquitinCre+ or *Hif1a^loxP/loxP^* UbiquitinCre+ mice (UbiquitinCre+ *n* = 12; *Hif1a^loxP/loxP^* UbiquitinCre+ *n* = 8 presented as mean ± SD; compared by Student’s *t*-test).

Previous studies have implicated HIF1A in cardioprotection from ischemia and reperfusion injury (5, 23, 38). For example, mice with Hif1a heterozygote deletion are not protected by ischemic preconditioning (39). Similarly, pharmacologic stabilizers of HIF, such as DMOG, provide robust cardioprotection during ischemia and reperfusion (5). However, the fact that mice with Hif1a homozygous deletion die during embryogenesis (40) has made it difficult to provide direct genetic evidence for *Hif1a* during cardioprotection. Thus, we generated mice with induced global deletion of *Hif1a* after tamoxifen treatment by crossing *Hif1a^loxP/loxP^* mice with Ubiquitin Cre+ mice to generate *Hif1a^loxP/loxP^* Ubiquitin Cre+ mice (41). To achieve global-induced *Hif1a* deletion, mice were treated daily with tamoxifen for 5 days (1 mg i.p./day) and recovered for 7 days before myocardial ischemia and reperfusion injury, using a previously described *in situ* model (Figure 1A) (34). Western blot analysis for Hif1a showed the expression of the Hif1a protein in response to Hif1a stabilizer treatment (DMOG, 1 mg i.p.) in Ubiquitin Cre+ mice. On the contrary, the Hif1a protein did not accumulate in cardiac tissue in *Hif1a^loxP/loxP^* Ubiquitin Cre+ mice after DMOG treatment (Figure 1B), suggesting a successful deletion of Hif1a in these animals.

To assess the functional consequences of global-induced deletion of Hif1a in myocardial ischemia and reperfusion injury, we exposed *Hif1a^loxP/loxP^* Ubiquitin Cre+ mice to *in situ* myocardial ischemia and reperfusion after treatment and recovery with tamoxifen (Figure 1A) and evaluated myocardial injury by infarct sizes and serum troponin levels. These studies demonstrated significantly larger myocardial infarct sizes after 60 min of ischemia and 2 h of reperfusion in *Hif1a^loxP/loxP^* Ubiquitin-Cre+ mice compared to littermate control Ubiquitin Cre+ mice after tamoxifen treatment (Figures 1C,D). Similarly, *Hif1a^loxP/loxP^* Ubiquitin Cre+ mice experienced significantly elevated of the cardiac injury marker troponin I (Figure 1E). Together, these studies demonstrate that Hif1a induced deletion is associated with dramatic increases in myocardial injury after ischemia and reperfusion, and confirm previous studies implicating HIF1A in cardioprotection (5, 38).

### Tissue-specific *Hif1a deletion* reveals a selective role for myeloid-derived Hif1A in mediating cardioprotection during ischemia and reperfusion

**FIGURE 2:**
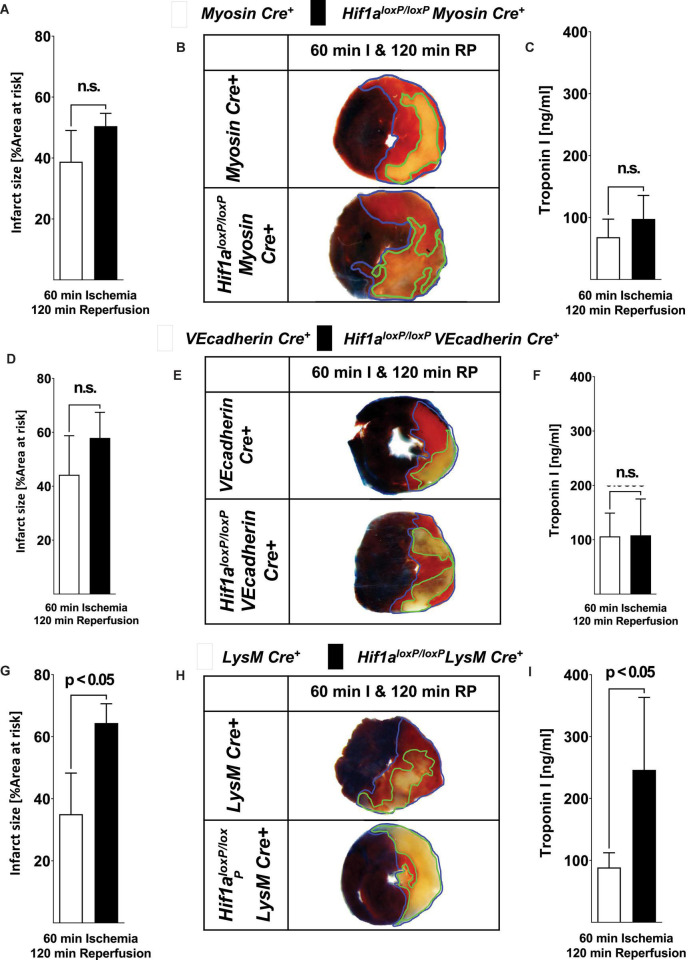
Myocardial ischemia and reperfusion injury in mice with tissue-specific Hif1a deletion in cardiac myocytes, vascular endothelia, or myeloid inflammatory cells. **(A–I)** *Hif1a^loxP/loxP^* were crossed with mice expressing Cre-recombinase under the control of heavy chain myosin promoter including a tamoxifen inducer (*Hif1a^loxP/loxP^* Myosin Cre+ mice) (23), vascular endothelial cadherin (*Hif1a^loxP/loxP^* VEcadherin Cre+ mice) (27). (*Hif1a^loxP/loxP^* VEcadherin Cre+) or lysozyme 2 (*Hif1a^loxP/loxP^* LysM Cre+ mice) (42) to generate tissue-specific HIF1A-deficient animals. Mice underwent 60 min of myocardial ischemia, followed by 120 min of reperfusion. Myocardial injury was determined by area at risk and troponin I serum concentration in *Hif1a^loxP/loxP^* Myosin Cre+ mice and age, sex, and weighed Myosin Cre+ mice who were matched received 1 mg of i.p. tamoxifen per day for 5 days followed by 7 days of recovery prior to experimentation. Infarct sizes ± SD in Myosin Cre+ or *Hif1a^loxP/loxP^* Myosin Cre+ presented as a percentage to the area-at-risk after 60 min of ischemia, followed by 120 min of reperfusion (Myosin Cre+ *n* = 6; *Hif1a^loxP/loxP^* Myosin Cre+ *n* = 4). **(B)** Representative infarct staining of Myosin Cre+ or *Hif1a^loxP/loxP^* Myosin Cre+ (60 min of ischemia and 120 min reperfusion). **(C)** Serum levels of troponin after 60 min ischemia, followed by 120 min of reperfusion in Myosin Cre+ or *Hif1a^loxP/loxP^* Myosin Cre+ (Myosin Cre+ *n* = 5; *Hif1a^loxP/loxP^* Myosin Cre+ *n* = 5). **(D)** Infarct sizes ± SD in VEcadherin Cre+ or *Hif1a^loxP/loxP^* VEcadherin Cre+ presented as percentage to the area-at-risk after 60 min of ischemia, followed by 120 min of reperfusion (VEcadherin Cre+ *n* = 7; *Hif1a^loxP/loxP^* VEcadherin Cre+ *n* = 7). **(E)** Representative infarct staining from VEcadherin Cre+ or *Hif1a^loxP/loxP^* VEcadherin Cre+ (60 min of ischemia and 120 min reperfusion). **(F)** Serum levels of Troponin after 60 min ischemia, followed by 120 min of reperfusion in VEcadherin Cre+ or *Hif1a^loxP/loxP^* VEcadherin Cre+ (VEcadherin Cre+ *n* = 4; *Hif1a^loxP/loxP^* VEcadherin Cre+ *n* = 6). **(G)** Infarct sizes ± SD in LysM Cre+ or *Hif1a^loxP/loxP^* LysM Cre+ presented as percentage to the area-at-risk after 60 min of ischemia, followed by 120 min of reperfusion (LysM Cre+ *n* = 8; *Hif1a^loxP/loxP^* LysM Cre+ *n* = 4). **(H)** Representative infarct staining of LysM Cre+ or *Hif1a^loxP/loxP^* LysM Cre+ (60 min of ischemia and 120 min reperfusion). **(I)** Troponin serum levels after 60 min ischemia, followed by 120 min of reperfusion in LysM Cre+ or *Hif1a^loxP/loxP^* LysM Cre+ (LysM Cre+ *n* = 4; *Hif1a^loxP/loxP^* LysM Cre+ *n* = 3); (all values represent mean ± SD; statistical significance assessed by two-sided, unpaired Student’s *t*-test).

Based on the above studies showing that global-induced deletion of Hif1A has deleterious effects during *in situ* myocardial ischemia and reperfusion injury, we next pursued studies to identify tissue-specific contributions of *Hif1a* in cardioprotection. To do this, we generated mice with *Hif1a* deletion in different tissue compartments, including cardiomyocytes (*Hif1a^loxP/loxP^* Myosin Cre+ mice) (23), vascular endothelial cells (*Hif1a^loxP/loxP^* VEcadherin Cre+ mice) (27) or myeloid cells (*Hif1a^loxP/loxP^* LysM Cre+ mice) (42). We subjected these mouse lines with Cre+ control mice of the same sex, weight, and age to 60 min of myocardial ischemia and 120 min of reperfusion and measured myocardial injury by assessing the size area of the infarct and serum troponin levels, as previously (12, 23, 35, 43). Consistent with previous studies (23), we found that *Hif1a^loxP/loxP^* Myosin Cre+ mice had similar infarct sizes and troponin I levels compared to Myosin Cre+ controls (Figures 2A–C). Similarly, deletion of Hif1a in vascular endothelia (*Hif1a^loxP/loxP^* VEcadherin Cre+ mice) did not alter the susceptibility to myocardial injury (Figures 2D–F). Surprisingly, we found the predominant phenotype in mice with Hif1a deletion in the myeloid compartment. Indeed, *Hif1a^loxP/loxP^* LysM Cre+ mice had dramatic increases in myocardial infarct sizes, and increased troponin I leakage into the plasma, as compared to LysM Cre+ control animals (Figures 2G–I). Together, these studies provide novel evidence that mice with myeloid-specific *Hif1a* deletion experience increased susceptibility to myocardial ischemia and reperfusion injury, essentially resembling the findings in mice with induced global deletion of Hif1A (Figures 1C–E).

### Transfer of *Hif1a*-deficient polymorphonuclear neutrophils is associated with more severe myocardial ischemia and reperfusion injury

**FIGURE 3:**
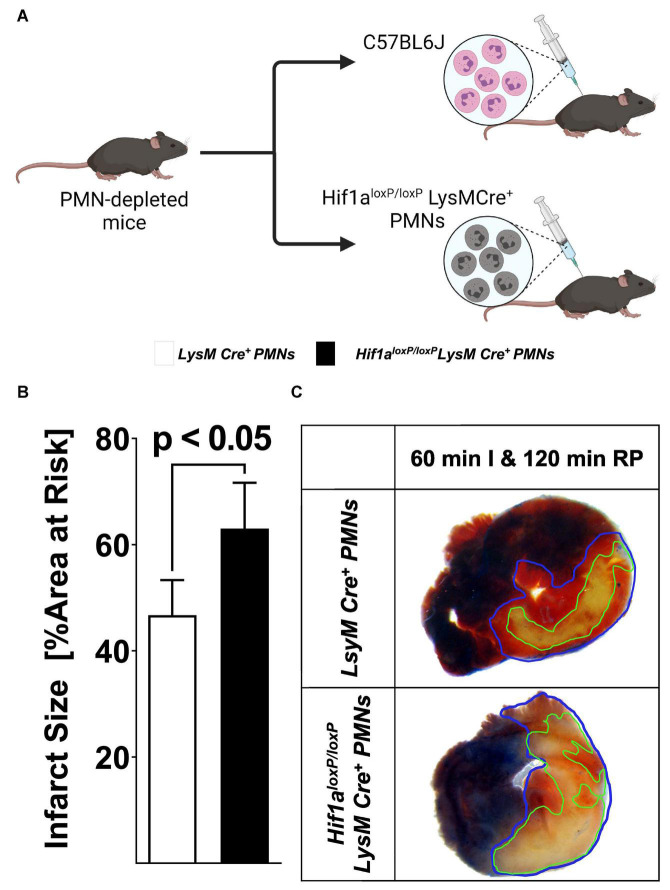
Effects of adoptive transfer of polymorphonuclear neutrophils (PMN) from *Hif1a^loxP/loxP^* LysM Cre+ to wild-type mice during myocardial ischemia and reperfusion injury. **(A)** Schematic of adoptive transfer model. PMNs deficient in C57BL6J or Hif1a-deficient PMNs (*Hif1a^loxP/loxP^* LysM Cre+) were isolated by negative selection and transferred to PMN-depleted C57BL6 animals (1A8 Ly6G specific antibody 24 h prior to experiment). The animals were then subjected to 60 min of ischemia, followed by 120 min of reperfusion with assessment of myocardial injury. **(B)** Infarct sizes ± SD in mice receiving PMN from C57BL6J or *Hif1a^loxP/loxP^* LysM Cre+ mice presented as a percentage of the area at risk after 60 min of ischemia, followed by 120 min of reperfusion (LysM Cre+ PMNs *n* = 4; *Hif1a^loxP/loxP^* LysM Cre+ *n* = 4; all values represented as mean ± SD; statistical significance evaluated by two-sided, unpaired Student’s *t*-test). **(C)** Representative infarct staining of neutrophil-depleted mice receiving PMNs from LysM Cre+ or *Hif1a^loxP/loxP^* LysM Cre+ mice.

The above studies indicate myeloid-dependent HIF1A in cardio-protection from ischemia and reperfusion injury. As next step, we pursued studies to further characterize specific cellular populations that are critical in this response. Due to the fact that PMNs are the predominant bone marrow-derived inflammatory cells in the post-ischemic myocardium (36), we next performed studies to address PMN-dependent HIF1A. For this purpose, we performed an adoptive transfer as described previously (43). In short, C57BL6J animals received 250 μg of 1A8 Ly6G-antibody 24 h prior to experimentation to deplete PMNs. On the day of the experiment, we isolated a pure population of mature PMNs from *Hif1a^loxP/loxP^* LysM Cre+ or matched wild-type control mice and infused *via* an intravascular catheter into PMN-depleted animals followed by 60 min of myocardial ischemia followed by 2 h of reperfusion (Figure 3A). In line with a functional role of HIF1A expressed in PMNs, we found that reconstitution with *Hif1a*-deficient PMNs was associated with dramatically increased infarcts sizes, when compared to mice reconstituted with control PMNs (Figures 3B,C), resembling myocardial injury phenotypes in *Hif1a^loxP/loxP^* LysM Cre+ mice. Together, these results indicate that PMN-dependent Hif1a deficiency is associated with elevated myocardial ischemia and reperfusion injury and implicates PMN-dependent HIF1A in cardioprotection.

### Pharmacologic stabilization of hypoxia inducible factor in isolated polymorphonuclear neutrophils prior to myocardial injury confers cardioprotection

**FIGURE 4:**
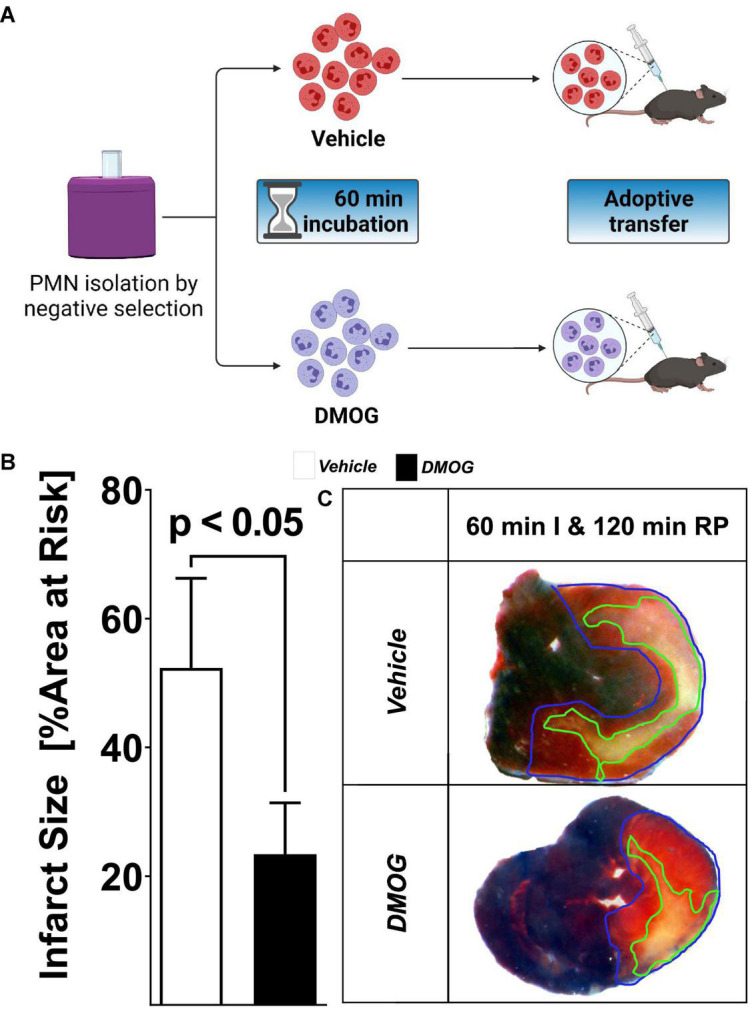
Effect of HIF stabilization in PMN on myocardial injury. **(A)** Schematic of the pharmacological stabilization of HIF in PMN. Cells were harvested by negative selection from C57BL6 mice; isolated cells were incubated for 60 min in vehicle or 1 mM DMOG solution. The cells were then adoptively transferred to PMN-depleted C57BL6, treated with 1A8 Ly6G-specific antibody 24 h before the experiment. **(B)** Infarct sizes ± SD in mice receiving vehicle or DMOG treated PMNs presented as percentage to the area-at-risk after 60 min of ischemia, followed by 120 min of reperfusion (vehicle *n* = 5; DMOG *n* = 4; all values represented as mean ± SD; statistical significance assessed by two-sided, unpaired Student’s *t*-test). **(C)** Representative infarct staining from neutrophil-depleted mice receiving PMN treated with DMOG or vehicle.

Based on the above studies showing that reconstitution with Hif1A-deficient PMNs prior to myocardial injury is associated with more severe myocardial infarction after ischemia and reperfusion, we next pursued opposite studies using PMNs treated *ex vivo* with a pharmacologic HIF activator. For this purpose, we used the HIF stabilizer DMOG. Previous studies from our laboratory had shown that systemic treatment with DMOG provides cardioprotection *via* stabilization of HIF1A (5, 23, 38). Similar to the experimental approach above, we first pursued PMN depletion prior to the experimentation by injecting mice with 250 μg of 1A8 Ly6G-antibody 24 h prior to myocardial ischemia and reperfusion. We isolated PMNs from C57BL6J mice and subsequently exposed PMNs for 60 min to vehicle or 1 mM DMOG (44). After the incubation period, we performed an adoptive transfer into PMN-depleted mice followed by 60 min of *in situ* ischemia and 120 min of reperfusion (Figure 4A). Mice that had received PMNs pre-treated with DMOG had significantly attenuated myocardial injury as compared to vehicle controls (Figures 4B,C). Taken together, these studies highlight that adoptive transfer of *ex vivo* DMOG treated PMNs before myocardial ischemia and reperfusion is associated with attenuated reperfusion injury and suggests a functional role of PMN-dependent HIF in cardioprotection.

### Selective role of myeloid HIF1A deficiency in mediating the cardioprotective effects of prolylhydroxylase-inhibitor treatment dimethyloxalylglycine

**FIGURE 5:**
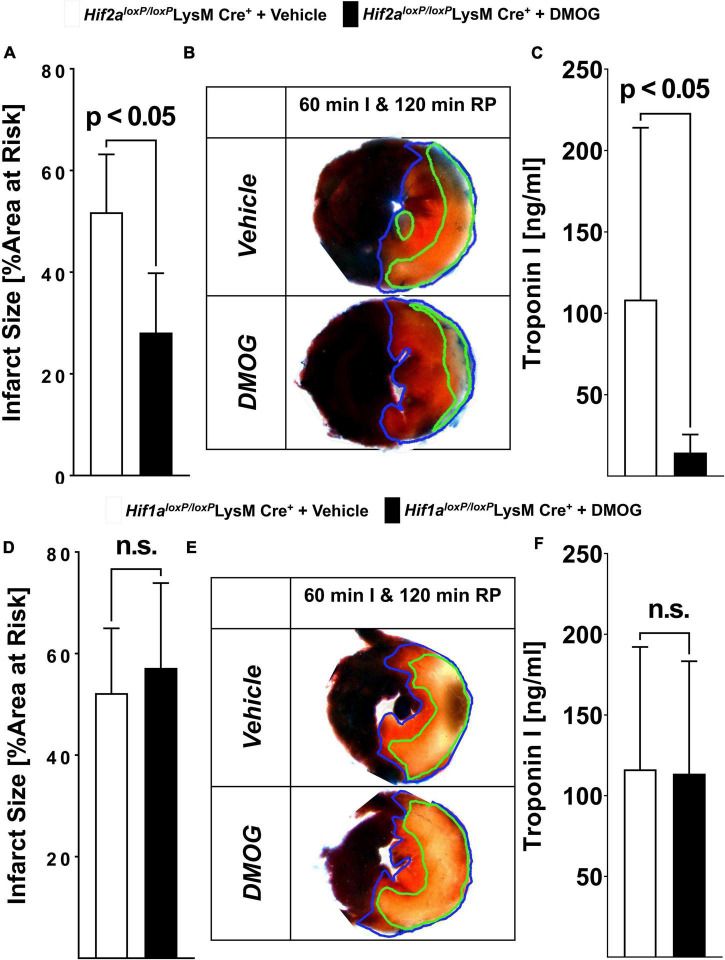
Selective HIF-isoform deletion in PMN in myocardial ischemia and reperfusion. **(A–F)** *Hif1a^loxP/loxP^* or *Hif2a^loxP/loxP^* were crossed with Cre recombinase expressing mice under the control of the lysozyme 2 promoter (LysM Cre+) to generate animals deficient in myeloids-specific Hif1a or Hif2a. Mice received vehicle or 1 mg of HIF stabilizing DMOG 4 h before ischemia. Mice underwent 60 min of ischemia and 120 min of reperfusion and evaluation of myocardial injury infarct sizes measurement and serum troponin I concentrations. **(A)** Infarct sizes were determined in vehicle or DMOG treated *Hif2a^loxP/loxP^* LysM Cre+ mice (*n* = 5 per group). **(B)** Representative infarct staining from vehicle or DMOG treated *Hif2a^loxP/loxP^* LysM Cre+ mice. **(C)** Serum levels of troponin in vehicle or DMOG treated *Hif2a^loxP/loxP^* LysM Cre+ mice (*n* = 5 per group). **(D)** Infarct sizes were determined in vehicle or DMOG treated *Hif1a^loxP/loxP^* LysM Cre+ mice (*Hif1a^loxP/loxP^* LysM Cre+ + vehicle *n* = 5; *Hif1a^loxP/loxP^* LysM Cre+ + DMOG *n* = 6). **(E)** Representative infarct staining from vehicle or DMOG treated *Hif1a^loxP/loxP^* LysM Cre+ mice. **(F)** Serum levels of troponin in vehicle or DMOG treated *Hif1a^loxP/loxP^* LysM Cre+ mice (*Hif1a^loxP/loxP^* LysM Cre+ + vehicle *n* = 5; *Hif1a^loxP/loxP^* LysM Cre+ + DMOG *n* = 6) (All data presented as mean ± SD. Statistical significance assessed by two-sided, unpaired Student’s *t*-test).

Previous studies have implicated both isoforms of HIFA (HIF1A or HIF2A) in cardio-protection from ischemia and reperfusion injury, including protective effects mediated by treatment with HIF stabilizers such as DMOG (5, 23, 35). To gain insight into the relative contributions of myeloid-expressed *Hif1a* or *Hif2a* in mediating DMOG elicited cardioprotection, we compared *Hif1a^loxP/loxP^* LysM Cre+ or *Hif2a^loxP/loxP^* LysM Cre+ on their capacity for infarct size reduction provided by pre-treatment with DMOG. In these studies, *Hif1a^loxP/loxP^* LysM Cre+, or *Hif2a^loxP/loxP^* LysM Cre+ were pre-treated with 1 mg DMOG i.p. or vehicle control 4 h before myocardial ischemia. Subsequently, animals were exposed to 60 min of myocardial ischemia and 120 min of reperfusion. In line with a functional role of myeloid-expressed Hif1a in mediating DMOG-protection we found that *Hif2a^loxP/loxP^* LysM Cre+ mice are responsive to DMOG pre-treatment with reduced ischemia and reperfusion injury compared to vehicle, as demonstrated in myocardial infarct sizes and troponin I-leakage (Figures 5A–C). In contrast, we found that *Hif1a^loxP/loxP^* LysM Cre+ failed to respond to pre-treatment with DMOG. Myocardial infarct size of *Hif1a^loxP/loxP^* LysM Cre+ pre-treated with DMOG were similar to that of *Hif1a^loxP/loxP^* LysM Cre+ pretreated with vehicle control (Figures 5D–F). Taken together, these results indicate that myeloid HIF1A as opposed to myeloid HIF2A is required for the cardio-protection provided by PHD inhibitors. In conjunction with our studies showing attenuated infarct sizes after *ex vivo* treatment with HIF stabilizer, these findings provide additional data implicating PMN-dependent HIF1A in cardio-protection from ischemia and reperfusion injury.

### Identification of neuronal guidance molecule netrin-1 as polymorphonuclear neutrophil-dependent Hif1A target critical for hypoxia inducible factor-dependent cardioprotection

**FIGURE 6:**
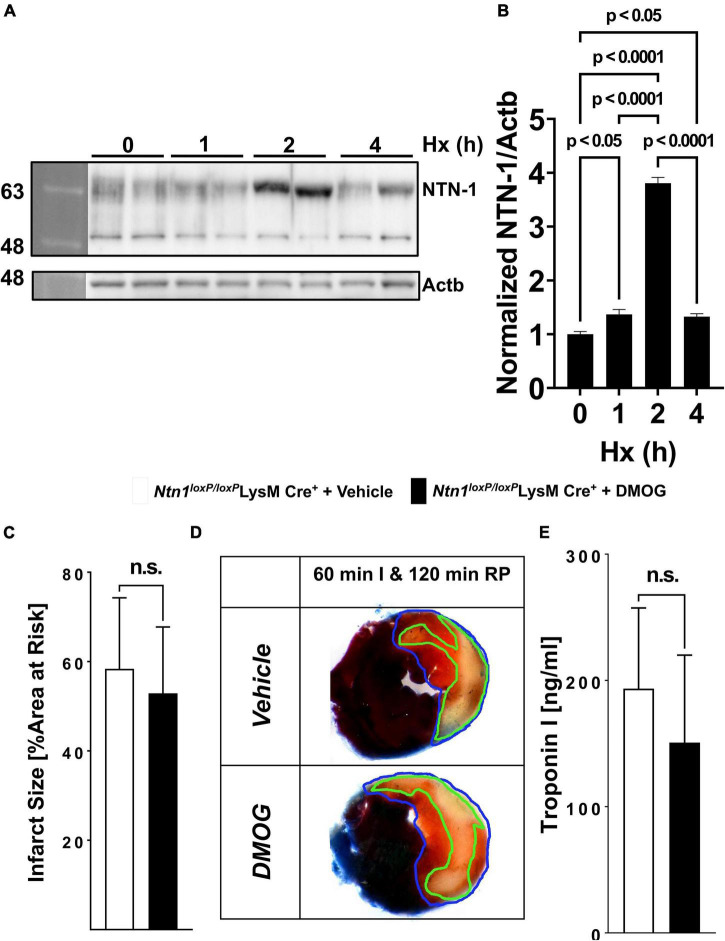
Netrin-1 as a PMN-dependent Hif1A target critical for HIF-dependent cardioprotection. **(A,B)** Isolated human PMNs from healthy donors underwent hypoxia for 0, 2 and 4 h followed by cell lysis and total protein harvest. **(A)** Immunoblot for NTN1. b-Actin (Actb) served as the loading control. One representative blot of three experiments is shown. **(B)** Quantitation by densitometry of the NTN1 immunoblot results relative to b-Actin (*n* = 2 per group; per group mean ± SD; compared by one-way ANOVA followed by Tukey’s multiple comparison test). **(C–E)** *Ntn-1*^loxP/loxP^* mice* were crossed with Cre recombinase-expressing mice under the control of the lysozyme 2 promoter (LysM Cre+) to generate mice deficient in myeloid-specific Ntn-1. Mice received vehicle or 1 mg DMOG 4 h prior to ischemia, then underwent 60 min of ischemia followed by 120 min of reperfusion and assessment of myocardial injury. **(C)** Infarct sizes were determined in vehicle or DMOG treated Ntn-1loxP/loxP LysM Cre+ (*Ntn-1*^loxP/loxP^** LysM Cre+ with vehicle *n* = 5; *Ntn-1*^loxP/loxP^** LysM Cre+ with DMOG *n* = 6). **(D)** Representative infarct staining from vehicle or DMOG treated *Ntn-1*^loxP/loxP^** LysM Cre+. **(E)** Troponin serum levels of vehicle or DMOG treated *Ntn-1*^loxP/loxP^** LysM Cre+ mice (*Ntn-1*^loxP/loxP^** LysM Cre+ with vehicle *n* = 5; *Ntn-1*^loxP/loxP^** LysM Cre+ with DMOG *n* = 6). All data are presented as mean ± SD. Statistical significance assessed by two-sided, unpaired Student’s *t*-test. DMOG-induced cardioprotection was absent in *Ntn-1*^loxP/loxP^** LysM Cre+.

Based on the above studies demonstrating functional roles of neutrophil-dependent HIF1A in cardioprotection from ischemia and reperfusion, we subsequently pursued studies to identify a transcriptional target in PMNs that would account for these observations. Recent studies from our laboratory had shown that PMN-derived netrin-1 functions to attenuate *in situ* myocardial ischemia and reperfusion injury. Netrin-1 was originally described as a neuronal guidance molecule important in brain development (45). However, more recent studies implicate netrin in orchestrating inflammatory events, including myocardial inflammation (46). Moreover, other studies had implicated HIF1A in the transcriptional regulation of netrin-1 during hypoxia, and identified netrin-1 as a classic HIF-target gene (42, 47). To examine a functional role of hypoxia-signaling in inducing PMN-dependent netrin-1, we first examined expression of netrin-1 during condition of ambient hypoxia. For this purpose, we freshly isolated human PMNs from peripheral blood and submitted the cells to 0, 2, and 4 h of ambient hypoxia (2% oxygen) and probed the cell lysates for netrin-1 expression by Western blot. Consistent with previous studies showing induction of netrin-1 expression during hypoxia (47), we found a very robust induction of PMN-dependent netrin-1 during ambient hypoxia (Figures 6A,B). Recent studies from our laboratory demonstrated the mice with deletion of myeloid-expressed netrin-1 (*Ntn1^loxP/loxP^* LysM Cre+ mice) experience increased myocardial infarct sizes and show that elevated levels of netrin-1 during myocardial infarction predominantly stems from PMNs (12). To further establish a link between the HIF-pathway and myeloid-dependent netrin-1 during cardioprotection, we next pursued studies with the pharmacologic HIF stabilizer DMOG. Here, we hypothesized that if myeloid-dependent HIF functions through induction of netrin-1 to provide cardioprotection, the cardioprotective effects of DMOG would be abolished in *Ntn1^loxP/loxP^* LysM Cre+ mice. To address this hypothesis, we treated *Ntn1^loxP/loxP^* LysM Cre+ mice with DMOG (1 mg i.p. 4 h prior to myocardial ischemia) or vehicle control, a treatment protocol that we had previously shown to be effective in reducing myocardial infarct sizes (5, 23). Consistent with a functional role of PMN-dependent netrin-1 in mediating DMOG-dependent cardioprotection, we found that the previously shown reduction of myocardial infarct sizes associated with DMOG treatment (Figures 5A–C) (5, 23) were completely abolished in *Ntn1^loxP/loxP^* LysM Cre+ mice (Figures 6C–E). Together with recent studies from our laboratory showing that *Ntn1^loxP/loxP^* LysM Cre+ mice experience increased infarct sizes (12), these findings implicate HIF-dependent induction of netrin-1 in the cardioprotection provided by myeloid-expressed HIF1A during ischemia and reperfusion injury of the heart.

## Discussion

Hypoxia-inducible factors such as HIF1A have been implicated in tissue adaption during limited oxygen availability, inflammation, or ischemia and reperfusion injury (48). While previous studies have directly implicated HIF1a in limiting myocardial infarct sizes during ischemia and reperfusion injury (5, 38, 39, 49, 50), the relative contributions of HIF1A expressed in different cell types in the heart remain elusive. To investigate tissue-specific functions of HIF1A in cardioprotection from *in situ* ischemia and reperfusion injury, we took a stepwise approach. In initial studies, we used a genetic approach, where Hif1A deletion was induced in the adult mouse, thereby circumventing embryonic lethality of homozygote Hif1a deletion (40). These studies revealed dramatic increase of myocardial infarct sizes in mice with induced global Hif1A deletion (*Hif1a^loxP/loxP^* UbiquitinCre+ mice) as compared to Cre+ controls. As second step, we pursued studies of myocardial ischemia and reperfusion injury in mice with genetic deletion of *Hif1a* in different tissue compartments, including cardiomyocytes (*Hif1a^loxP/loxP^* Myosin Cre+ mice), vascular endothelial cells (*Hif1a^loxP/loxP^* VEcadherin Cre+ mice) or in myeloid inflammatory cells (*Hif1a^loxP/loxP^* LysM Cre+ mice). To our surprise, only mice with deletion of *Hif1a* in myeloid inflammatory cells resembled the previous findings we had established in mice with induced global deletion of Hif1A, thereby indirectly implicating myeloid-dependent *Hif1a* in cardio-protection from ischemia and reperfusion. Due to the critical functions and high presence of PMNs during myocardial reperfusion injury (51), we studied mice with reconstitution of wild-type or *Hif1a*-deficient neutrophils following antibody-mediate PMN depletion (52). While mice with *Hif1a*-deficient PMNs experience increased myocardial injury, mice with reconstitution of wild-type PMN that received *ex vivo* treatment with HIF stabilizer DMOG were protected. Finally, studies that focused on transcriptional targets of HIF1A in neutrophils implicated PMN-dependent netrin-1 in mediating HIF1A elicited cardioprotection. Together, these studies highlight a functional role of myeloid-dependent HIF1A in attenuating myocardial ischemia and reperfusion, and its transcriptional target netrin-1. Based on these studies, using HIF stabilizers or treatment with recombinant netrin-1 may represent novel approaches to treat or prevent myocardial injury during myocardial ischemia and reperfusion.

Netrin-1 improves the signaling events of the adenosine receptor Adora2b (53), which elicits strong anti-inflammatory effects, as previous studies have shown (47). In recent studies, netrin-1 was shown to require a functional Adora2b receptor on the surface of neutrophils to induce cardio protective effects (12). Likewise, Adora2b was previously identified as the target for hypoxia signaling through HIF1A. Studies of murine models of myocardial infarction and reperfusion injuries demonstrate the strong cardiovascular protection of Adora2b. A specific agonist for Adora2b significantly reduced the size of infarct after myocardial ischemia (5). Studies using the HIF activator DMOG as a treatment approach for experimental myocardial ischemia showed that cardioprotection was eliminated in mice lacking Adora2b (5). This directly links the signaling of HIF1A and ADORA2B during cardioprotection.

Adora2b reduces the size of the infarct due to its strong inhibition of the inflammatory response. This has been demonstrated in studies in which Adora2b deficiency in mice increased the expression of pro-inflammatory and cardiotoxic cytokine tumor necrosis factor alpha (TNF-α) (36). Netrin-1 has similar anti-inflammatory effects in hypoxic inflammation that require the presence of ADORA2B (47) – and both Netrin-1 and ADORA2B are HIF1a-target genes. This suggests that, in response to myocardial infarction and reperfusion, activation of HIF1A coordinates cardioprotective effects by activating the expressions of Netrin-1 and ADORA2B, which both reduce post-ischemic myocardial inflammation.

While the current studies directly implicate HIF1A in attenuating myocardial ischemia and reperfusion injury, other studies have also implicated HIF2A in cardioprotection. HIF2A is an isoform of HIF1A and several studies have identified either complementary or opposing functions to HIF1A (35, 54–57). As such, it is not surprising that previous studies have also identified a functional role of Hif2A in attenuating myocardial infarct sizes during ischemia and reperfusion injury (23). In contrast to the current studies that implicated myeloid-dependent Hif1A in cardioprotection, those studies demonstrated a functional role of HIF2A expressed in cardiac myocytes. In fact, *Hif2a^loxP/loxP^* Myosin Cre+ mice exhibited larger infarct sizes during *in situ* myocardial ischemia and reperfusion injury (23). Subsequent studies on myocyte-dependent HIF2A target genes implicate the epidermal growth factor amphiregulin (AREG) in mediating HIF2A dependent cardioprotection (23). Additional studies in myocardial biopsies of patients with ongoing ischemia revealed elevated levels of AREG (23), of the amphiregulin receptor ERBB1 (35). Mice with global deletion of Areg (*Areg^–/–^* mice), or with deletion of the *ErbB1* receptor expressed on cardiac myocytes (*ErbB1^loxP/loxP^* Myosin Cre+) experienced increased susceptibility to myocardial ischemia and reperfusion injury. Together, those studies implicate myocyte-dependent HIF2A in coordinating the induction and signaling events of AREG through the ERBB1 receptor in cardioprotection (23, 35). Together with the current studies, those findings accentuate a cardioprotective role of HIF, by attenuating myocardial ischemic injury through myocyte-dependent HIF2A and myocardial reperfusion injury through myeloid-dependent HIF1A.

While the current studies concentrate on myocardial ischemia and reperfusion injury, previous studies had also implicated HIF in promoting ischemic preconditioning of the heart. Ischemic preconditioning involves short episode of ischemia that confers protection to a subsequent myocardial infarction (58). Several previous studies have shown functional roles of HIF1A in mediating the tissue-protective effects of ischemic preconditioning, including studies to address tissue-specific functions of HIF1A. Initial studies demonstrated that mice with partial deletion of *Hif1a* (*Hif1a*^±^ mice) experienced a complete loss of cardioprotection from ischemic preconditioning (26). Similarly, mice with siRNA *in vivo* silencing of Hif1a demonstrated a lack of cardio-protection by ischemic preconditioning, while pre-treatment with the HIF stabilizer DMOG preconditioned the myocardium (5). Subsequent studies to address tissue-specific functions of HIF1A in ischemic preconditioning implicated HIF1A activity in vascular endothelia (50). Other studies found that deletion of *Hif1a* in cardiomyocytes (*Hif1a^loxP/loxP^* Myosin Cre+) also abolishes the cardio-protective effects of ischemic preconditioning (23). In contrast, mice with cardiomyocyte-specific deletion of *Hif2a* (*Hif2a^loxP/loxP^* Myosin Cre+) were protected by ischemic preconditioning (23). Interestingly, more recent studies also implicate HIF1A in mediating the cardioprotective effects of remote ischemic preconditioning, where repeated episodes of remote ischemia to a limb can produce protection from myocardial or acute kidney injury (59). These studies indicate that during remote ischemic preconditioning, HIF1A is stabilized, and transcriptionally induces the HIF target gene L-10 with subsequent IL-10 signaling as a mechanism of cardioprotection (49). Taken together, these studies provide evidence from multiple studies that HIF1A – as opposed to HIF2A – mediates the cardioprotective effects of direct or remote ischemic preconditioning (17, 60).

**FIGURE 7:**
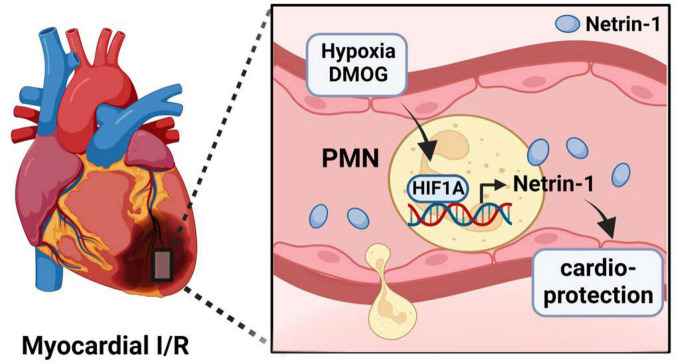
Suggested mechanism of HIF1A-induced cardioprotection during myocardial ischemia and reperfusion injury. During myocardial ischemia and reperfusion injury, neutrophils undergo hypoxia, leading to stabilization of HIF1A. HIF1A subsequently induces increased expression and release of neuronal guidance proteins Netrin-1 by PMN. Netrin-1 then reduces a reduction in infarct sizes.

The current studies implicate the HIF1A target netrin-1 in PMNs as critical target for mediating the cardioprotective effects of myeloid HIF1A. Netrin-1 is a neuronal guidance molecule that was initially described as a diffusible axon outgrowth promoting factor in nematodes (61). However, it became apparent that netrin-1 is much more ubiquitously expressed (62). In addition to its function as neuronal guidance molecule during brain development (45), it became apparent that netrin-1 can also function to modulate inflammatory endpoints or ischemia and reperfusion injury (63, 64). For example, netrin-1 released from the vagal nerve has been shown to attenuate excessive inflammation and promote the resolution of injury (64). Several previous studies have linked netrin-1 signaling in cardioprotection from ischemia and reperfusion injury, for example *via* interaction of netrin-1 with the classic netrin-1 receptor deleted in colorectal cancer DCC expressed on endothelial cells (65, 66). Consistent with current findings, a recent study indicates that PMN-dependent netrin-1 can function to provide cardioprotection during *in situ* ischemia and reperfusion injury by enhancing extracellular adenosine signaling events through the Adora2b adenosine receptor (12, 53, 63, 67, 68). Although extracellular adenosine signaling was initially described to induce a transient slowing of heart rate (69), many subsequent studies have sown functional roles for extracellular adenosine signaling in attenuating myocardial ischemia and reperfusion injury, including studies directly implicating myeloid Adora2b signaling (7, 36, 43) (Figure 7). Taken together, these studies provide additional support for the concept that myeloid HIF1A provides cardioprotection through transcriptional induction of its target gene netrin-1, leading to attenuated myocardial ischemia and reperfusion injury.

The current findings have important translational implications. For example, it is possible that patients experiencing myocardial ischemia and reperfusion injury could be treated with recombinant netrin-1 to activate the HIF1A-netrin-1 pathway for cardioprotection. Several other studies have shown that recombinant netrin-1 treatment can function to dampen myocardial injury (12) or other states of inflammatory disease (67). However, an alternative treatment approach would include the use of pharmacological HIF stabilizers. Over the past decade, several pharmaceutical companies have developed pharmacologic HIF activators (17, 18, 70–72). These pharmacological HIF activators function by inhibiting prolylhydroxylases and thus prevent the degradation of HIF1A *via* the proteasomal pathway (3). Pharmacological HIF activators, such as roxadustat or vadadustat, have been successfully tested for the treatment of renal anemia and phase 3 clinical trials and are available as oral medications (19–22). These orally available HIF activators could be used in patients experiencing myocardial ischemia and reperfusion injury. Furthermore, it is possible that these medications could be given to patients at high risk of experiencing myocardial injury, such as patients undergoing cardiac surgery. Treatment with an oral HIF activator would likely cause stabilization of HIF, including neutrophils, and thus dampen myocardial ischemia and reperfusion injury (73). This highlights that HIF1A coordinates the transcriptional response in response to myocardial ischemia.

## Data availability statement

The raw data supporting the conclusions of this article will be made available by the authors, without undue reservation.

## Ethics statement

This animal study was reviewed and approved by the University of Colorado Denver and the Institutional Animal Care and Use Committee of the University of Texas Health Science Center at Houston.

## Author contributions

KH-S, JL, and WR performed the experiments. XY and YW helped with data analysis. MK designed the study, performed the experiments and data analysis, and wrote the manuscript. HE designed the study and wrote the manuscript. All authors contributed to the article and approved the submitted version.
